# Dynamic Imaging of Aortic Pathologies: Review of Clinical Applications and Imaging Protocols

**DOI:** 10.14797/mdcvj.1172

**Published:** 2023-03-07

**Authors:** Peter Osztrogonacz, Marton Berczeli, Ponraj Chinnadurai, Su-Min Chang, Dipan J. Shah, Alan B. Lumsden

**Affiliations:** 1 Houston Methodist Hospital, Houston, Texas, US; 2 Semmelweis University, Budapest, Hungary; 3 Siemens Medical Solutions USA Inc., Malvern, Pennsylvania, US

**Keywords:** dynamic imaging, dynamic CTA, dynamic MRA, 4D flow MRI, aorta, aortic imaging, diagnostic imaging

## Abstract

The past decade has seen significant advances in dynamic imaging of the aorta. Today’s vascular surgeons have the opportunity to choose from a wide array of imaging modalities to evaluate different aortic pathologies. While vascular ultrasound and aortography are considered to be the bread and butter imaging modalities, newer dynamic imaging techniques provide time-resolved information in various aortic pathologies. However, despite growing evidence of their advantages in the literature, they have not been routinely adopted. In order to understand the role of these emerging modalities, one must understand their principles, advantages, and limitations in the context of various clinical scenarios. In this review, we provide an overview of dynamic imaging techniques for aortic pathologies and describe various dynamic computed tomography and magnetic resonance imaging protocols, clinical applications, and potential future directions.

## Introduction

Dynamic imaging is a rapidly evolving yet not fully adopted technique in the realm of medical imaging modalities. Most aortic pathologies have a dynamic component, such as aortic dissection, and any imaging technique must be nimble enough to capture this dynamic nature—not only to enable better diagnosis but also to aid in determining optimal treatment options. To account for cardiac motion-related artifacts, especially in the ascending aorta and aortic arch, an electrocardiogram (ECG)-gated imaging protocol typically is performed. The word “dynamic” in dynamic imaging can be broadly interpreted as introducing time as the fourth dimension to evaluate the dynamics of contrast enhancement in the aorta—for example, time-resolved angiography. In addition, such novel dynamic imaging techniques can enable one to assess and quantify the direction, velocity, and volume of blood flow to impact clinical decision-making, patient management, and optimal post-interventional surveillance.

Most cardiovascular imaging modalities have a dynamic component, including vascular ultrasound with contrast enhanced ultrasound, echocardiography, intravascular ultrasound, x-ray angiography, dynamic computed tomography angiography (d-CTA), and dynamic magnetic resonance angiography (MRA), such as 4-dimensional (4D) flow magnetic resonance imaging (MRI).

Although each of the above-mentioned modalities can provide “dynamic” information during the evaluation of aortic pathologies, we limit the scope of this article to discussing the three most commonly used noninvasive dynamic 3D cross-sectional imaging techniques: dynamic CTA, dynamic MRA, and 4D flow MRI. Herein we provide an overview of dynamic aortic imaging techniques, describing various CT/MRI protocols, clinical applications, and potential future prospects.

## Imaging Modalities and Protocols

### Dynamic CT Angiography

**Figure 1:**
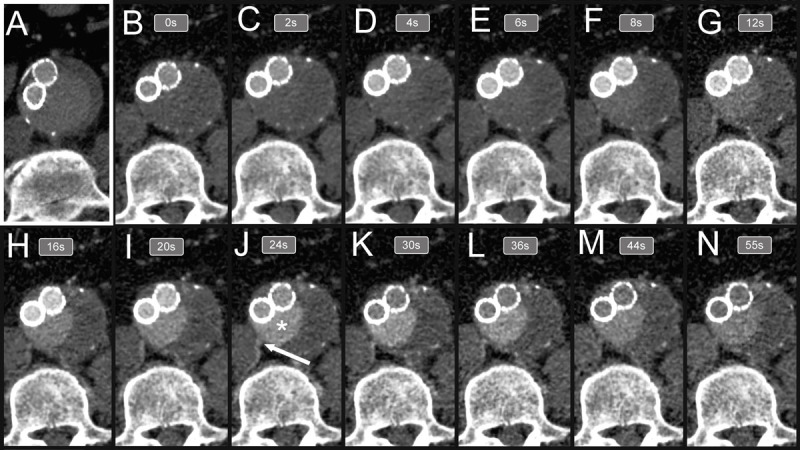
Delayed phase computed tomography (CTA) and dynamic CTA (d-CTA) images across multiple timepoints. During d-CTA, approximately 10 to 15 images are acquired in different timepoints after the contrast bolus to assess the exact type of endoleak. In this particular case, the most informative image is acquired around 24 seconds after contrast injections (panel J). The aneurysm sac (white * sign) is filled by the right lumbar artery (white arrow). In comparison, the image acquired with standard CTA in the delayed phase falls behind the acquired d-CTA images in terms of identifying the source of endoleak.

**Figure 2:**
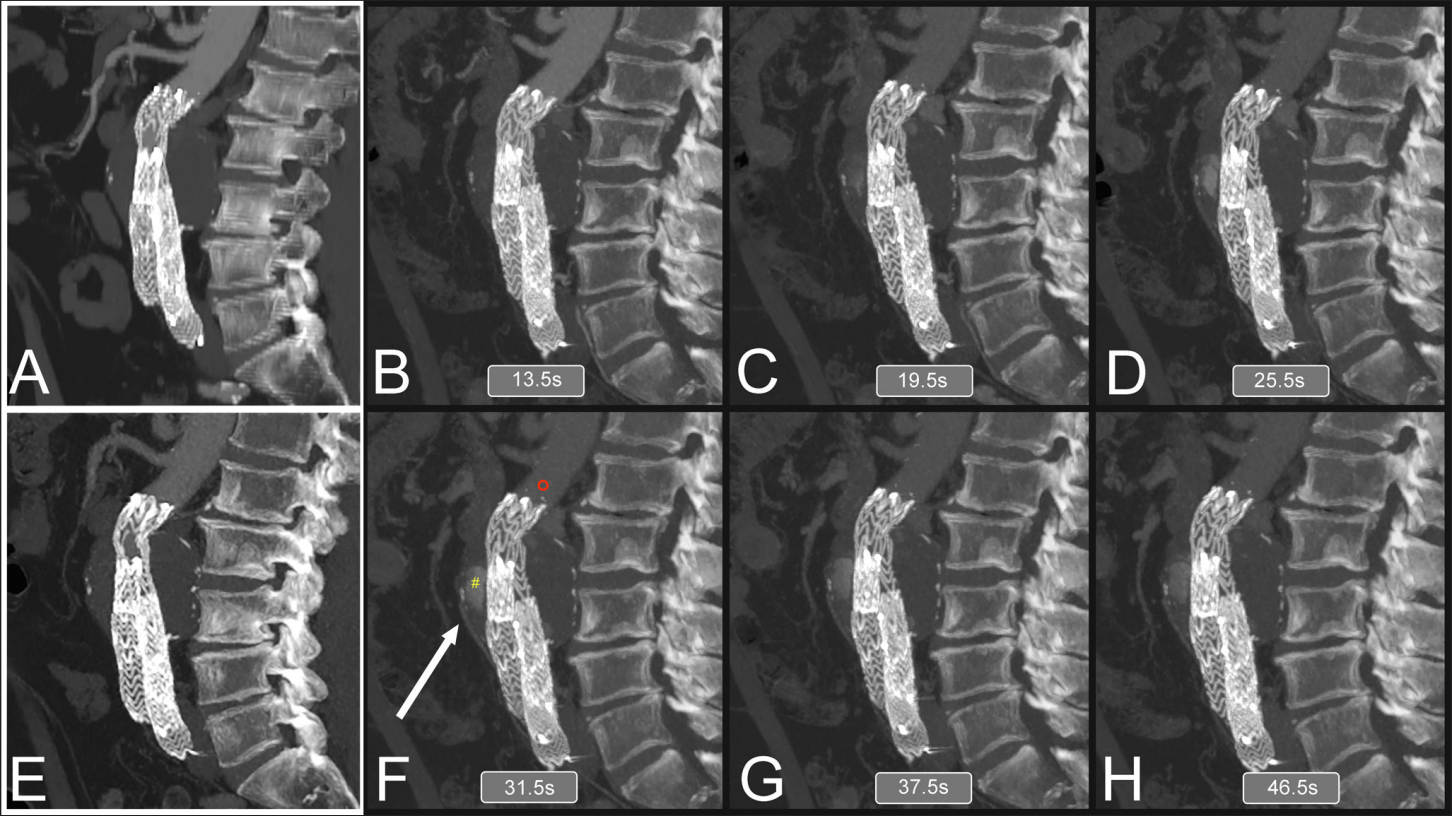
Comparison of arterial and delayed phases of triphasic computed tomography (CTA) with dynamic CTA findings. Compared to triphasic CTA, dynamic CTA is superior in identifying endoleak following endovascular aneurysm repair (EVAR). In this particular case, neither the arterial (A) nor the delayed (2 E) CTA phases were able to support endoleak as the underlying cause of progressive aneurysm sac growth following EVAR. In contrast, dynamic endoleak (panels B-D, F-H) was capable of showing the endoleak and the supplying inferior mesenteric artery (panel F, white arrow).

**Figure 3:**
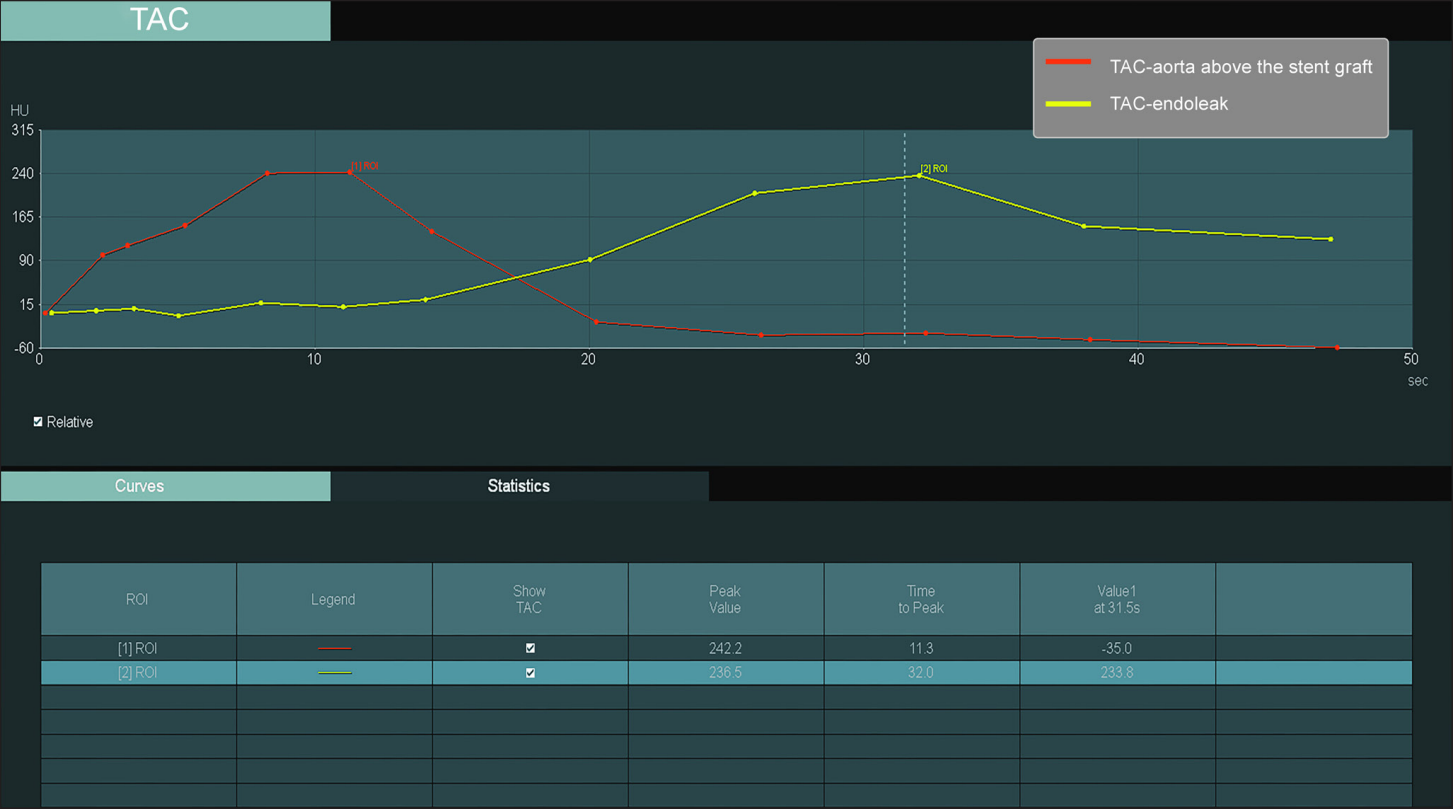
Time attenuation curve (TAC) of the aorta and the site of endoleak pertaining to Figure 2 dynamic CTA (d-CTA). The TAC of the d-CTA shows a significant delay (20 seconds) in the peak of the Hounsfield unit between the region of interest (ROI) placed in the aorta (red ° sign, ROI-1) and ROI placed at the site of the endoleak (yellow # sign, ROI-2), suggesting the sac filling from the inferior mesenteric artery toward the aneurysm sac rather than direct filling from the aorta.

Like conventional CTA imaging, d-CTA involves acquiring a series of CTA datasets (approximately 5-12 scans) across different points of the contrast enhancement curve after a bolus of iodinated contrast injection (Figures 1, 2, 3).^1^ In addition, ECG-gated CTA can be used to evaluate cardiac, ascending aortic, and arch pathologies by acquiring a series of CTA datasets (approximately10 scans) across different points of the cardiac cycle.^2^ These two techniques also can be combined to provide time-resolved contrast kinetics and ECG gating for cardiac motion, especially in the setting of post endograft surveillance in the ascending aorta and aortic arch.^3^ The technical details and our institutional CT aortic imaging protocols are summarized in Table 1.

**Table 1:** Dynamic computed tomography angiography imaging protocol for aortic endoleak detection. CT: computed tomography; CTA: computed tomography angiography; d-CTA: dynamic CTA


DYNAMIC CTA IMAGING PROTOCOL FOR AORTIC ENDOLEAK		

	STEPS	DETAILED STEPS

1	Patient positioning	Supine

2	Peripheral vein access	Right/left upper extremity

3	Topogram and noncontrast CT imaging acquisition	Sn-100 T in filter to reduce radiation exposure

4	Timing bolus to check contrast arrival time	10-20 mL contrast injection followed by 50 mL saline at 3.5-4 mL/min flow rate, region of interest in aorta

5	Distribution and scan number planning	Choose DynMulti4D menu from the pop-up menu, plan distribution and scan number based on timing bolus and findings on previous studies

6	Optimize imaging parameters	To reduce exposure, optimize kV, scan range, etc.

7	Contrast injection for d-CTA acquisition	70-80 mL contrast injection followed by 100 mL saline at 3.5-4 mL/min flow rate

8	Start d-CTA acquisition	Use delay time determined by the timing bolus

9	Parameters for d-CTA scan	10-12 scans, tube voltage: 70-100 kV, tube current: 150 mAs, rotation time: 0.25 s, scan duration: 39 s, slice thickness: 0.7-1 mm, field of view (z-axis) : 23-33 cm


### Dynamic Magnetic Resonance Imaging

**Figure 4:**
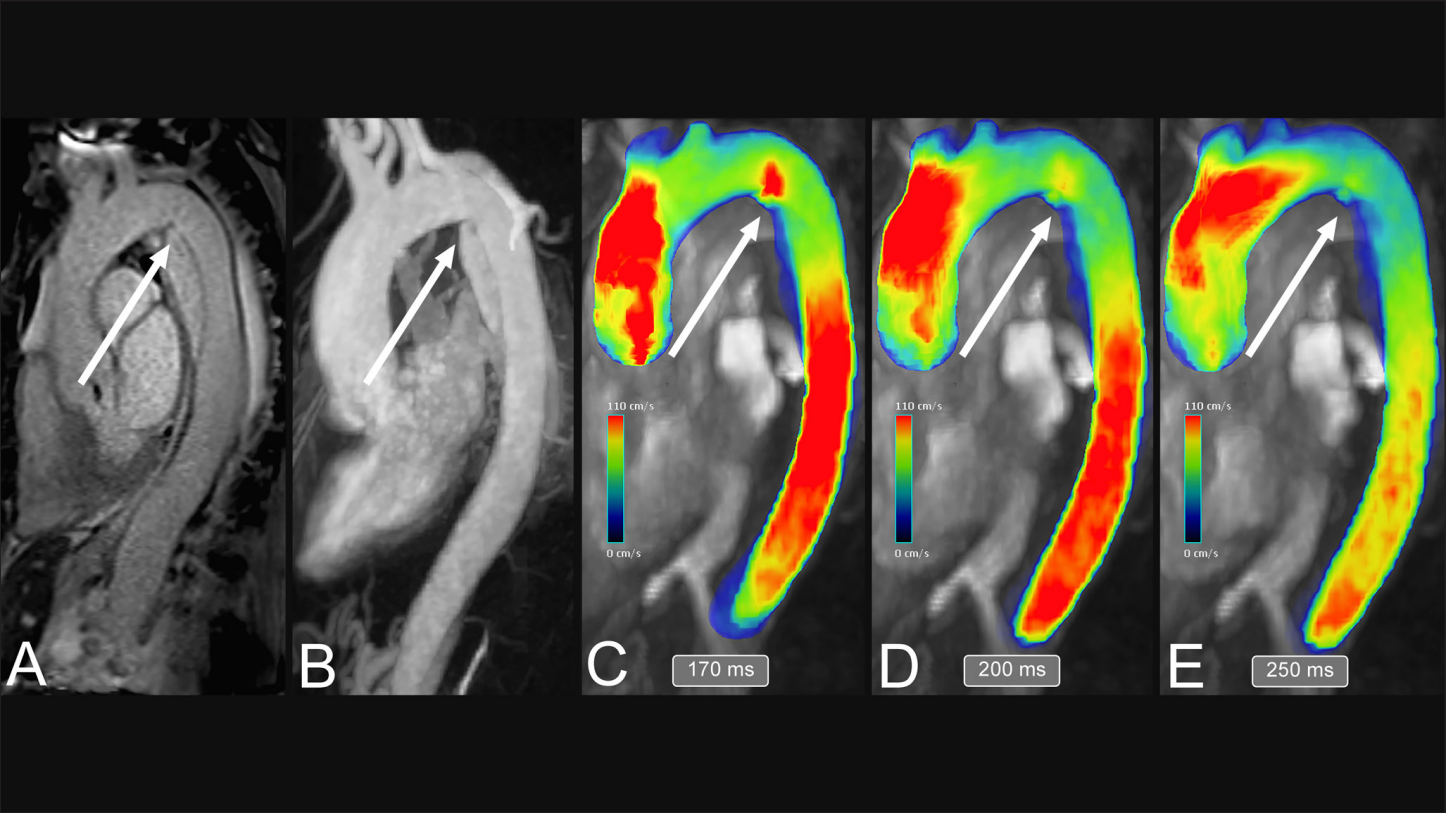
Qualitative analysis of type-B aortic dissection using magnetic resonance imaging (MRI). The contrast-enhanced MRA sequence (A) gives information on the site of the primary entry tear (white arrows), while the TWIST sequence shows the intimal flap and the directionality of false lumen filling (B). 4D flow MRI sequence (C-E) may complete the assessment of aortic dissection with color-coded velocity mapping, which is potentially able to uncover the tear where the false lumen is filling through (white arrows, C-E).

**Figure 5:**
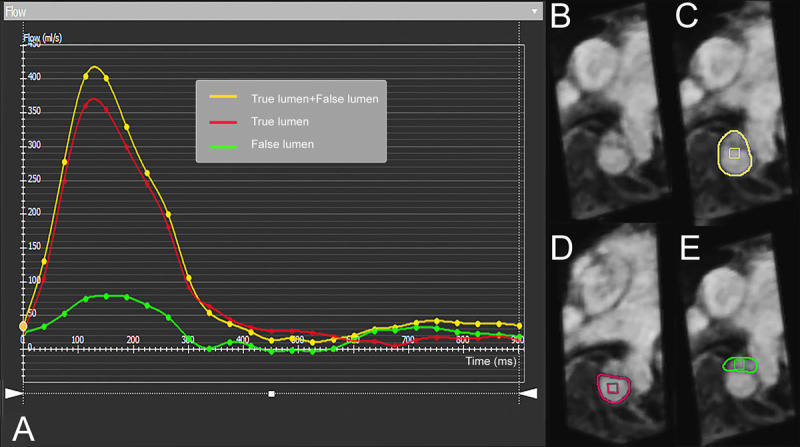
Quantitative flow evaluation of true lumen and false lumen using 4-dimensional (4D) flow magnetic resonance imaging (MRI). 4D flow MRI provides quantification of flow parameters depicted by the marked areas on panels B-E. The flow volume is displayed in a graph (A) where the y-axis represents the flow volume (mL/s) through the marked area and the x-axis represents time in milliseconds. In aortic dissection, the true lumen (A: red line, D: red outline) and the false lumen (A: green line, E: green outline) can be interrogated, and the entire flow volume (A: yellow line, C: yellow outline) can be determined.

Unlike CTA imaging, MRI can provide better insights into aortic pathologies by imaging vessel wall characteristics without any additional radiation exposure. However, its clinical utilization is still evolving due to relatively longer acquisition times, the need for a dedicated setup, a steeper learning curve, and understanding and optimizing multiple MR imaging sequences. The technical details and our institutional MR aortic imaging protocols are summarized in Table 2. In addition to conventional imaging sequences, the 4D flow MRI sequence deserves a special mention because it is a time-resolved 3D phase contrast MRI sequence capable of providing quantitative and qualitative flow information across any segment of the aortic lumen (Figures 4, 5). It does not require any additional contrast agent, although having an on-board contrast agent increases the signal-to-noise ratio.

**Table 2:** Dynamic magnetic resonance imaging protocol. MRI: magnetic resonance imaging; DICOM: Digital Imaging and Communications in Medicine; HASTE: half-fourier acquisition single-shot turbo spin-echo; PACS: picture archiving and communication system; SSFP: steady-state free precession; TWIST: time-resolved angiography with stochastic trajectories; VIBE: volumetric interpolated breath-hold examination


DYNAMIC MRA PROTOCOL		

	STEPS	DETAILED STEPS

1	Patient positioning	Supine

2	Peripheral vein access	Right/left upper extremity

3	Acquiring mask or later subtraction	Chest-abdomen noncontrast full-resolution image

4	Contrast administration	Gadolinium: 30-40 mL contrast material + 50 mL saline injection to wash out the contrast agent from the periphery Ferumoxytol (feraheme): 3 mL with 27 mL saline followed by 50 mL saline flush

5	Contrast passage visualization	Done by acquiring scan

6	Regular sequences are acquired for dynamic MRI scan	SSFP cine to evaluate dynamic vs static obstruction in TBAD HASTE, bright blood SSFP, 3D contrast-enhanced MRA to evaluate aortic morphology, TWIST for time resolved MRA, T1 VIBE for steady state imaging, 4D flow for qualitative and quantitative flow evaluation

7	4D flow MRI	VENC 150-200 cm/s, acquisition time 7-12 min, free breathing

8	Send acquired data to database	Acquired data is sent to the institutional cloud-based imaging database (PACS)

9	Image analysis	Using DICOM viewer connected to the institutional PACS


### Clinical Applications of Dynamic Aortic Imaging

Aortic pathologies cover a broad spectrum of vascular diseases of the aorta, spanning from the aortic valve to the distal abdominal aorta. In this section, we provide a descriptive overview of clinical applications of the dynamic imaging technique across various aortic pathologies and its impact on treatment planning and guidance based on our early clinical experience.

#### 1. AORTIC Valvular Disease

In developed countries, aortic stenosis (AS) ranks as the most common valvular heart disease, occurring in 12.4% of elderly patients.^4,5^ Transthoracic echo (TTE) is considered to be the mainstay of diagnosis and classification of disease severity, providing information on peak jet velocity, mean transvalvular pressure gradient, and aortic valve area. Contrast-enhanced CTA imaging is also a standard-of-care imaging technique to facilitate better aortic morphometry and plan for potential transcatheter therapies. In the setting of AS, cardiac MRI is performed in select situations to study any other associated cardiac anomalies and to provide better noninvasive hemodynamic information. Adriaans et al.^6^ compared TTE and 4D flow and found the latter to improve concordance between AS parameters regarding peak jet velocity, mean transvalvular pressure gradient, and aortic valve area. The study suggested the potential of 4D flow MRI as a comprehensive noninvasive quantitative technique, especially in patients with inconsistent findings on TTE.

Aortic regurgitation (AR) can occur with or without AS, and TTE is the gold standard modality for diagnosis and evaluation based on current guidelines.^7,8^ CTA adds the anatomical information required for invasive treatment planning. In addition, cardiac MRI is considered to be a complementary imaging technique, although it gives more accurate information on left ventricle volume, mass and function as well as aortic dilatation compared with TTE.^9,10^

#### 2. Acute Aortic Syndrome

Acute aortic syndrome is an umbrella term referring to aortic dissection, intramural hematoma, and penetrating aortic ulcer. All three conditions are considered potentially lethal and may overlap with each other.

Since acute type A dissection presents as an imminent life-threatening condition, MRI currently is not considered an option for preoperative planning given its time-consuming nature, nor is dynamic CTA due to its limited field of view.

##### 2.1 Type-B Aortic Dissection

Type-B aortic dissection (TBAD) occurs when a tear develops in the intimal layer distal to the origin of the innominate artery,^11^ and blood flow enters the space between the intimal and medial layers of the aorta, creating true and false lumen throughout the length of the affected aortic segment. The intimal flap may compromise blood supply via the aortic branches by causing either dynamic or static obstruction. In general, standard operative strategy for complicated TBAD includes primary entry tear coverage with a stent graft to facilitate false lumen collapse and positive aortic remodeling, although dynamic imaging may uncover a more distal tear as the one supplying the false lumen. Failure of culprit fenestration coverage could lead to subsequent interventions, increasing the risk for perioperative morbidity such as access-related complications, progression of aortic dissection, or spinal cord ischemia. Moreover, dynamic imaging could also aid in discerning between dynamic and static obstruction of visceral vessels, consequently affecting operative planning. While d-CTA is capable of the latter, given the limited field of view, it does not allow for imaging of the entire aorta, which excludes d-CTA from initial TBAD assessment. In fact, it can be preserved for follow-up imaging to address specific questions, such as determining residual static versus dynamic occlusion of the aortic branches.

Overall, several studies have been published regarding 4D flow MRI in TBAD patients, yet each of them lacks long-term follow-up and an appropriate number of cases.^12,13,14,15,16^ Apparently, 4D flow MRI seems comparable to triphasic CTA in terms of fenestration detection in TBAD patients based on the study conducted by Allen et al.^12^ Another study concluded that 4D flow MRI image quality is comparable to CTA for TBAD assessment.^13^ A third paper evaluated false lumen pressure analysis in chronic TBAD patients and identified false lumen ejection fraction and absolute pressure difference between distal false lumen and aortic root as independent factors for aortic growth.^14^ Ruiz-Muñoz et al. examined false lumen flow dynamics and biomechanics and identified in-plane rotational flow, pulse wave velocity, and false lumen thrombus as factors positively related to aortic growth in chronic aortic dissection patients.^16^

On the whole, the concept of 4D flow MRI imaging in TBAD patients seems to be established (Figure 4); however, further longitudinal large cohort studies are required to assess its place in TBAD management.

Dynamic MRA could be helpful to determine the flow directionality in the false lumen, but it does not provide additional information compared with 4D flow MRI. Kinner et al. compared dynamic MRA (TWIST sequence) to high-resolution contrast-enhanced MRA in acute aortic dissection patients. On one hand, they have found the latter as significantly more sensitive and even comparable to CTA in terms of image quality.^17^ On the other hand, dynamic MRA affected the treatment plan for 10 out of 19 patients, which underlines the importance of dynamic imaging in this particular aortic pathology (Figure 5).

##### 2.2 Intramural Hematoma and Penetrating Aortic Ulcer

Intramural hematoma (IMH) occurs when blood enters the intimal/medial layer without visible entry tear. Penetrating aortic ulcer (PAU) develops in the aortic wall as a focal outpouching to the internal elastic lamina due to atherosclerotic plaque erosion and inflammatory changes.^18,19^

In general MRI is an excellent modality to assess and follow IMH and PAU lesions,^20,21^ however the role of dynamic imaging is not yet established in the setting of these types of aortic pathologies. Although in theory dynamic imaging could provide additional information on the feeding vessel and blood pool in cases of acute/subacute IMH, further research is necessary to acquire sufficient evidence-based data.

IMH is a dynamic phenomenon, thus addressing it with time-resolved imaging can help to better identify influencing factors. IMH has several imaging features, such as intramural blood pools, which are considered a benign prognostic factor and may favor conservative management.^22^ Berczeli et al. recently highlighted that d-CTA imaging can help in understanding the pathophysiology behind these blood pools by identifying flow direction and their source.^23^

#### 3. Aortic Coarctation

Aortic coarctation (CoA) is a congenital cardiovascular condition in which the aorta is narrowed, most commonly around the level of the ligamentum arteriosum.^24^ CoA is responsible for 5% to 7% of congenital heart disease cases and predisposes to hypertension, aortic insufficiency, aortic dissection, and aortic aneurysm.^25,26^ Based on the location of the narrowing, CoA is classified as preductal, ductal, or postductal, the latter being the most common type in adults.^27^ Treatment is based on pressure gradient through the stenosis and post-stenotic pressure loss.^28^ When the systolic pressure gradient exceeds 20 mm Hg, intervention is recommended according to current guidelines.^26^ The pressure gradient could be measured either by invasive catheterization or by TTE. 4D flow MRI offers a noninvasive alternative to measure flow proximal and distal to the coarctation in all three dimensions, addressing the shortcomings of the previous two modalities, namely invasiveness in case of catheterization and the lack of time-resolved 3D information on part of TTE. Additionally, 4D flow MRI provides information about the collateral flow as well.^29^ Riesenkampff et al. compared 4D flow MRI and catheterization, in terms of pressure measurement at 5 different locations on the aorta and concluded a good agreement between the two modalities, suggesting that 4D flow MRI could potentially replace invasive catheterization in the evaluation of CoA.^26^

Overall 4D flow MRI is a promising technique regarding CoA assessment, which may guide the perioperative management of patients with CoA in the future.

#### 4. Thoracic and Thoracoabdominal Aneurysms

The term “aneurysm” refers to a localized dilation involving all 3 layers of the vessel, which exceeds the normal caliber by more than 150%. The most common thoracic aortic aneurysms are located on the ascending aorta (40%), followed closely by descending thoracic aortic aneurysms (35%). The aortic arch (15%) and the thoracoabdominal localization (10%) are considered less frequent. Due to recent advancements in aortic graft development, in specific scenarios, the ascending aortic stent graft implantation offers a feasible alternative to open repair. However, follow-up of these grafts presents a challenge with traditional d-CTA, given the frequent occurrence of cardiac motion related artefacts. In these instances, dynamic electrocardiography-gated CTA offers a viable solution and optimizes imaging sensitivity for endoleak detection.^3^ The descending thoracic and abdominal aorta have not been studied extensively in terms of dynamic imaging, to date. Based on the data published on 4D flow MRI performed on ascending aortas, wall shear stress and oscillatory shear index (instantaneous WSS fluctuation) play an important role in ascending aorta dilation.^30,31,32^ These findings are probably applicable to the descending thoracic aorta, although further studies are required to explore the role of 4D flow MRI at the level of the descending thoracic aorta.

#### 5. Abdominal Aortic Aneurysm

The infrarenal aorta is considered the most common predilection location for aneurysmal disease.^33^ Endovascular aneurysm repair (EVAR) is the mainstay of infrarenal abdominal aortic aneurysm (AAA) treatment due to its superior perioperative survival rate compared to open surgical repair,^34,35,36,37^ type II endoleak (T2EL) being the most common endoleak following EVAR procedures.^38^ T2EL is caused by retrograde filling of the aneurysm sac after stent graft implantation, usually from lumbar branches and/or the inferior mesenteric artery (IMA). Although dynamic imaging may not provide additional information compared to triphasic CTA and MRA in the preoperative evaluation of infrarenal AAAs, it presents a unique option during the course of postoperative follow-up in terms of diagnosing and differentiating between the various types of endoleaks and determining the culprit vessel(s) feeding the aneurysm sac in the setting of T2EL.^39^

While triphasic CTA is able to assess aneurysm sac growth and suggests the type of endoleak, d-CTA has been reported as more accurate compared to triphasic CTA in terms of identifying the type of endoleak following an EVAR procedure.^40,41,42^ Additionally, Berczeli et al.^42^ demonstrated the applicability of d-CTA in intraoperative image guidance as well, underlining the versatility of this particular imaging modality. However, as tempting as it seems to perform d-CTA for routine follow-up after EVAR, in our day-to-day practice we tend to reserve it for cases where findings on triphasic CTA are inconclusive regarding the origin of the endoleak (Figures 1, 2).

4D flow MRI offers an alternative to d-CTA for endoleak detection following EVAR. Although 4D flow MRI is considered inferior compared to traditional CTA in terms of spatial resolution, it can be improved with contrast administration.^43^ The combined functional and anatomical information acquired this way can aid in endoleak detection, treatment planning, and even in intraoperative image guidance. Sakata et al.^44^ compared CTA and 4D flow MRI in endoleak detection and found 4D flow MRI to be more sensitive in terms of endoleak identification.

In fact, 4D flow MRI provides a plethora of information on intraluminal flow patterns, which could be used as predictors for certain aortic conditions, including aneurysm sac growth in T2EL. Flow analysis may contribute to a better understanding of T2EL and helps to identify the patient group that could benefit from T2EL repair.

A recent retrospective single-center study used 4D flow MRI to identify parameters related to aneurysm sac growth in the setting of T2EL. First, they found higher initial peak flow velocity and amplitude of dynamics of blood flow in the persistent feeding vessel group after 1 year. Second, they found a correlation between aneurysm sac expansion and initial higher amplitude of dynamics of blood flow.^45^ Van der Laan et al.^46^ examined MRI plus dynamic MRA and found it to be superior to MRI in endoleak detection, although further studies have not been conducted for this application of dynamic MRI.

High-resolution MRA combined with 4D flow MRI has an advantage over dynamic CTA by being able to image the whole aorta with no radiation burden while allowing for the detection of flow patterns and wall shear stress. However, the sensitivity of 4D flow MRI in T2EL detection is yet to be determined.

#### 6. Aortic Occlusion

Although no data currently exists in the literature on dynamic imaging in aortic occlusion patients, 4D flow MRI could potentially allow for quantitative collateral flow assessment in chronic aortic occlusion to determine the necessity or extent of aortic reconstruction. In comparison, dynamic CTA and dynamic MRA may not be suitable for functional assessment of aortic occlusion since they do not provide an opportunity for in-depth flow analysis.

## Future Directions

Although vascular ultrasound and digital subtraction angiography are the most frequently used dynamic imaging tools, these modalities lack 3D visualization. In contrast, dynamic CTA, dynamic MRA, and 4D flow MRI offer 3D-rendered time-resolved alternatives. Obviously, each of these modalities have disadvantages, but the majority of them have already been addressed. For example, dynamic CTA is associated with a moderate amount of radiation burden, which could potentially be significantly reduced in the future with the latest generation CT scanners (for example, photon-counting CT technology). Also, 4D flow MRI acquisition must become faster and more accurate to offer an alternative to CTA in acute cases and to reach widespread recognition. Additionally, the general knowledge of this modality must be established to take full advantage of it. One approach to these challenges could be the introduction of compressed sensing, more robust automated segmentation, and utilization of computational fluid dynamics with adaptive mesh refinement.^47^

Currently, dynamic imaging in general is used sporadically without a uniform disease-tailored protocol, which is natural given the lack of large cohort longitudinal trials. Therefore, more large-scale studies are required to establish evidence in dynamic imaging of the aorta. Time-resolved cross-sectional imaging modalities carry great potential and may open the door to a new era in vascular surgery, where we can offer patient-tailored treatment for a variety of aortic pathologies to an extent never seen before.

## Key Points

- Dynamic computed tomography angiography (CTA), dynamic magnetic resonance angiography (MRA), and 4-dimensinal (4D) flow MRI are dynamic imaging techniques enabling 3D cross-sectional imaging of the aorta.
- Dynamic CTA is an established imaging technique that proved to be the most suitable for identification and classification of endoleaks during post endograft surveillance.
- Although the routine clinical utilization of dynamic MRA and 4D flow MRI in aortic conditions is still evolving, they carry an enormous potential by allowing for flow pattern analysis along the entire aorta.
- The capability to image the entire aorta and provide qualitative and quantitative flow analysis are the main advantages of dynamic MRA and 4D flow MRI over dynamic CTA, which operates with a limited field of view.
